# Gyrfalcon Prey Abundance and Their Habitat Associations in a Changing Arctic

**DOI:** 10.1002/ece3.70763

**Published:** 2025-01-08

**Authors:** Michaela Gustafson, Jennifer D. McCabe, Brian W. Rolek, Travis L. Booms, Michael T. Henderson, Leah Dunn, David L. Anderson, Jennyffer Cruz

**Affiliations:** ^1^ Department of Biological Sciences and Raptor Research Center Boise State University Boise Idaho USA; ^2^ The Peregrine Fund Boise Idaho USA; ^3^ Alaska Department of Fish and Game Fairbanks Alaska USA

**Keywords:** Arctic trophic web, habitat associations, predator–prey, prey abundance, raptors, territory occupancy

## Abstract

Arctic habitats are changing rapidly and altering trophic webs and ecosystem functioning. Understanding how species' abundances and distributions differ among Arctic habitats is important in predicting future species shifts and trophic‐web consequences. We aimed to determine the habitat–abundance relationships for three small herbivores on the Seward Peninsula of Alaska, USA by fitting data from 983 point counts (collected during 2019, 2021, and 2022) with N‐mixture models that account for imperfect detection. These herbivore species, Willow Ptarmigan ( *Lagopus lagopus* ), Rock Ptarmigan ( *L. muta* ), and Arctic ground squirrels ( *Urocitellus parryii)* , are fundamental to tundra food webs, and primary prey for Arctic raptors including Gyrfalcons ( *Falco rusticolus* ). Second, we aimed to map herbivore densities within Gyrfalcon breeding territories. Third, we aimed to evaluate whether Gyrfalcons were more likely to occupy territories with higher prey densities using a multi‐season occupancy model coupled with occupancy observations from helicopter surveys conducted during 2016–2022 at 97 Gyrfalcon territories. We found that male Willow Ptarmigan were more abundant in areas with greater cover of tundra, tall shrubs, and tussock tundra. Conversely, male Rock Ptarmigan were more abundant in areas with greater cover of sparse vegetation and tundra. Arctic ground squirrels were more abundant at higher elevations with greater cover of sparse vegetation and low shrubs. Willow Ptarmigan were widespread within Gyrfalcon breeding territories, whereas Rock Ptarmigan and Arctic ground squirrels had patchier distributions with few areas of high abundance. Lastly, Gyrfalcons were more likely to occupy territories with higher densities of Willow Ptarmigan and Arctic ground squirrels. As the Artic continues to warm, Rock Ptarmigan and Arctic ground squirrels may be vulnerable to ongoing shrub encroachment, whereas Willow Ptarmigan may benefit. By tying abundances of three prey to Gyrfalcon occupancy, our results contribute to understanding potential impacts on higher levels of this Arctic trophic web.

## Graphical abstract

Arctic habitats are changing rapidly, impacting trophic webs and ecosystem functions. We examined the habitat–abundance relationships of three small herbivores on the Seward Peninsula, Alaska, and their distribution within Gyrfalcon breeding territories. We found that Gyrfalcons preferred territories with higher densities of Willow Ptarmigan and Arctic ground squirrels, and that changes in habitat may benefit some prey species, like Willow Ptarmigan, while making others, such as Rock Ptarmigan and Arctic ground squirrels, more vulnerable to warming and shrub encroachment.

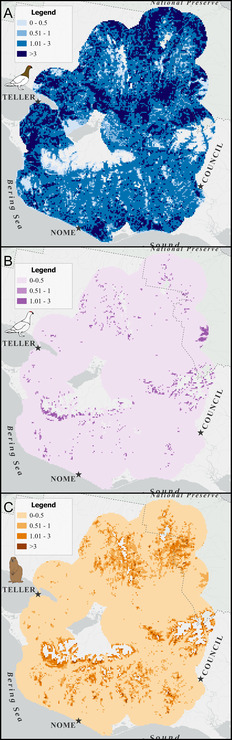

## 1 Introduction

Arctic ecosystems are characterized by unique trophic webs with distinctive and dynamic ecological interactions (Meltofte, Josefson, and Payer 2013; Schmidt et al. 2017). However, the Arctic is experiencing accelerated climate change, with increased precipitation (Bintanja and Selten 2014; McCrystall et al. 2021) and average temperatures rising four times faster than those at lower latitudes (Rantanen et al. 2022). These altered weather patterns are expected to have strong ecological effects (Hassol and Corell 2006). For example, open and tree‐less tundra habitats that are characteristic of this region are being invaded by tall shrubs and trees (Tape, Sturm, and Racine 2006; Myers‐Smith et al. 2011, 2015). Such changes are predicted to affect Arctic animal communities and species population dynamics (Gilg et al. 2012). Understanding associations between Arctic wildlife and their habitats will be essential to monitoring ecological change and conserving sensitive species (Verberk 2011).

Habitat and climatic changes in the Arctic are expected to alter the demography of species, causing declines or disappearances for some species and increases for others via habitat and range shifts (Gilg et al. 2012). Over half of Arctic‐breeding wading birds (Charadriiformes) are reported to be declining (Smith et al. 2020). These declines are partially attributed to environmental changes in the Arctic such as habitat loss and phenological mismatches (Kwon et al. 2019; Smith et al. 2020). Conversely, climate‐induced environmental changes are facilitating the northward expansion of some species into the Arctic. The increase in woody shrubs in the Arctic has allowed for the North American beaver ( *Castor canadensis* ) to expand its range to areas of the Arctic tundra (Tape et al. 2022). The presence of the beaver and its landscape‐altering behaviors have cascading effects that could be intensifying the effects of climate change in the Arctic (Tape et al. 2018). Knowledge about demographic responses to environmental changes provides early insights potentially useful for predicting range contractions or expansions and ensuring that conservation strategies can be implemented in a timely manner, thus preserving trophic webs unique to the Arctic (Matthews and Whittaker 2015).

Recent studies suggest that Willow Ptarmigan ( *Lagopus lagopus* ), Rock Ptarmigan ( *L. muta* ) and Arctic ground squirrels ( *Urocitellus parryii* ) have declined in parts of the Arctic, potentially driven by changes in climate and habitat. Comprehensive reviews show declining trends in Willow Ptarmigan populations across Fennoscandia (Lehikoinen et al. 2014; Fuglei et al. 2020) and eastern Russia (Fuglei et al. 2020), whereas a dampening of population cycles has been observed in the Yukon territory of Canada (Mossop 2011). Willow Ptarmigan declines in Finland have been correlated with more snow‐free spring days (Melin et al. 2020), willow thicket fragmentation from ungulate browsing (Henden et al. 2011; Ims and Henden 2012), increased winter temperatures, and the collapse of small rodent populations (Kausrud et al. 2008). However, climate change is predicted to have conflicting effects on Willow Ptarmigan populations because changes in temperature and precipitation could reduce breeding success, whereas shrub encroachment may provide additional habitat (Scridel et al. 2021). Conversely, the expansion of woody shrubs into tundra habitats is already documented and is expected to reduce the extent of suitable habitat for Rock Ptarmigan and Arctic ground squirrels. Rock Ptarmigan populations exhibit negative trends in Iceland and Greenland (Fuglei et al. 2020) and mainland Europe (Revermann et al. 2012; Imperio et al. 2013; Lehikoinen et al. 2014; Furrer et al. 2016; Canonne et al. 2020) and are predicted to experience a major loss (> 50%) of their alpine habitat to tree‐line and shrub encroachment and increasing temperatures (Revermann et al. 2012; Pernollet, Korner‐Nievergelt, and Jenni 2015; Ferrarini, Alatalo, and Gustin 2017; Hotta et al. 2019; Scridel et al. 2021). Arctic ground squirrel populations have decreased rapidly at lower elevations and been extirpated from some boreal forest habitats of Canada (Werner et al. 2015). Tall and dense vegetation impacts squirrels' ability to detect and evade predators, reducing suitability of such habitats to support viable populations (Gillis et al. 2005; Donker and Krebs 2012; Wheeler and Hik 2014a; Flower et al. 2019). Consequently, shrub encroachment has been suggested as one of the mechanisms leading to squirrel ( *Urocitellus parryii* ) declines (Wheeler and Hik 2013, 2014b; Wheeler et al. 2015).

The Gyrfalcon is an Arctic apex predator likely to be affected by bottom‐up effects caused by changes in prey abundance (Barichello and Mossop 2011; Booms, Lindgren, and Huettmann 2011). The largest of the true falcons (Nielsen and Cade 2017), they nest on bluffs and cliffsides along the waterways and mountainous terrain of the Arctic tundra. On the Seward Peninsula, Alaska, these cliffs occur in a dynamic landscape containing few roads, areas of isolated human development, as well as a national reserve. The nesting success and productivity of Gyrfalcons in this landscape is variable (Anderson et al. 2019; Henderson, Booms, et al. 2021) and some Gyrfalcon territories are occupied consistently while others are used only sporadically (Bente 2011; Anderson et al. 2019). This varied use may be related to prey availability (Sergio and Newton 2003). For Gyrfalcons on the Seward Peninsula, Willow Ptarmigan, Rock Ptarmigan, and Arctic ground squirrels compose most of their diet (Robinson et al. 2019; Johnson et al. 2022). Because raptors, as apex predators, frequently function as important signalers of ecosystem change (Natsukawa and Sergio 2022; Sergio et al. 2008; Sergio, Newton, and Marchesi 2008), clarifying factors that underly patterns of predator and prey distribution helps fill information gaps fundamental to modeling systemic changes in Arctic habitats.

Small Arctic herbivores remain understudied across much of their range despite their importance as ecosystem engineers (ptarmigan, Tape et al. 2010; Arctic ground squirrels, Wheeler and Hik 2013) and as primary prey in trophic webs. We aimed to fill this knowledge gap by associating different habitat types with landscape‐level abundances of Willow Ptarmigan, Rock Ptarmigan, and Arctic ground squirrels on the Seward Peninsula. Secondly, we aimed to estimate their spatial abundance and distribution within historically occupied Gyrfalcon breeding territories. Lastly, we used a multi‐season occupancy model to examine the relationship between density estimates of these three species and Gyrfalcon occupancy. Understanding spatial patterns of prey abundance, rather than presence, will help determine key habitats for these important Arctic species. Further, knowledge of prey abundance is important for explaining demographic differences among raptor territories (Newton 1979; Steenhof et al. 1999; Nielsen and Cade 2017; Anderson et al. 2019). By tying prey species abundances to Gyrfalcon occupancy, our results should contribute to future studies aimed at understanding the potential impacts of a changing Arctic on higher levels of this Arctic trophic web.

## 2 Methods

### 2.1 Study Area

The Seward Peninsula in western Alaska, ancestral land of the Iñupiat (Inupiaq and Yupik) People, is characterized by rugged, mountainous terrain flanked by rolling hills of Arctic tundra crossed by numerous streams and rivers. Dispersed rock outcroppings, inland cliffs, and cliff‐lined river systems provide nesting substrates for cliff‐nesting raptors, including the Gyrfalcon (Booms et al. 2010; Anderson et al. 2019). The climate is harsh with long, cold, and typically dry winters and short summers. Temperature extremes during the Gyrfalcon breeding season can range from −43°C in March to 30°C in July (NOAA n.d.).

The most widespread habitat on the Seward Peninsula is dwarf shrub meadow dominated by tussock forming sedges ( *Eriophorum vaginatum* and *Carex bigelowii* ) interspersed with varying densities of dwarf shrub, predominately *Betula nana* , with *Betula* and heath‐dominated communities in drier parts of the landscape (Kessel 1989). Major river drainages and protected foothill slopes are dominated by dense thickets of willow (*Salix* spp.) and alder (*Alnus* spp.; Kessel 1989). More exposed and windblown sites among the disconnected ridges, domes, and flat‐topped mountains are often barren or sparsely vegetated by dwarf shrub mat: prostrate vegetative communities varying in amounts of mosses, lichens, xeric herbs and forbs, and dwarf shrub (Kessel 1989).

The study area comprised 14,150 km^2^ of the southern portion of the Seward Peninsula bounded by the Bering Sea along the south and west, Niukluk and Solomon rivers to the east, and the Bering Land Bridge National Preserve to the north (Anderson et al. 2019). We conducted point count surveys within three corridors following the three roads present in the southern portion of the Seward Peninsula. We buffered each road segment with an 8‐km polygon on either side to maximize surveyor safety and survey efficiency. For safety reasons, we removed sections in the buffered roads that were inaccessible, such as sheer cliffs, and areas at elevations greater than 500 m, although we recognize that prey species occur above such elevations. We then divided each road segment buffer into 5 units of roughly equal area and placed an 800 m x 800 m grid over the units and roads. Next, we generated points at the vertices of the grid, creating points that were 800 m apart. Straight‐line distance from survey points to the nearest road ranged from 2.5 m to 7.95 km with 63 points (6.4%) occurring less than 200 m from the nearest road. We did not expect a road effect on point count surveys from species avoidance or distribution of vegetative communities near the roads because the roads are primitive (dirt roads often in poor condition), remote, and lightly traveled (Hutto et al. 1995). There is minimal human development and disruption to native vegetation adjacent to roads compared to vegetation further from the roadside (Wellicome et al. 2014). Additionally, McCarthy et al. (2012) and Lituma and Buehler (2016) respectively found negligible roadside bias in abundance and distribution of multiple species 200 and 0–600 m from the roadside.

**FIGURE 1:**
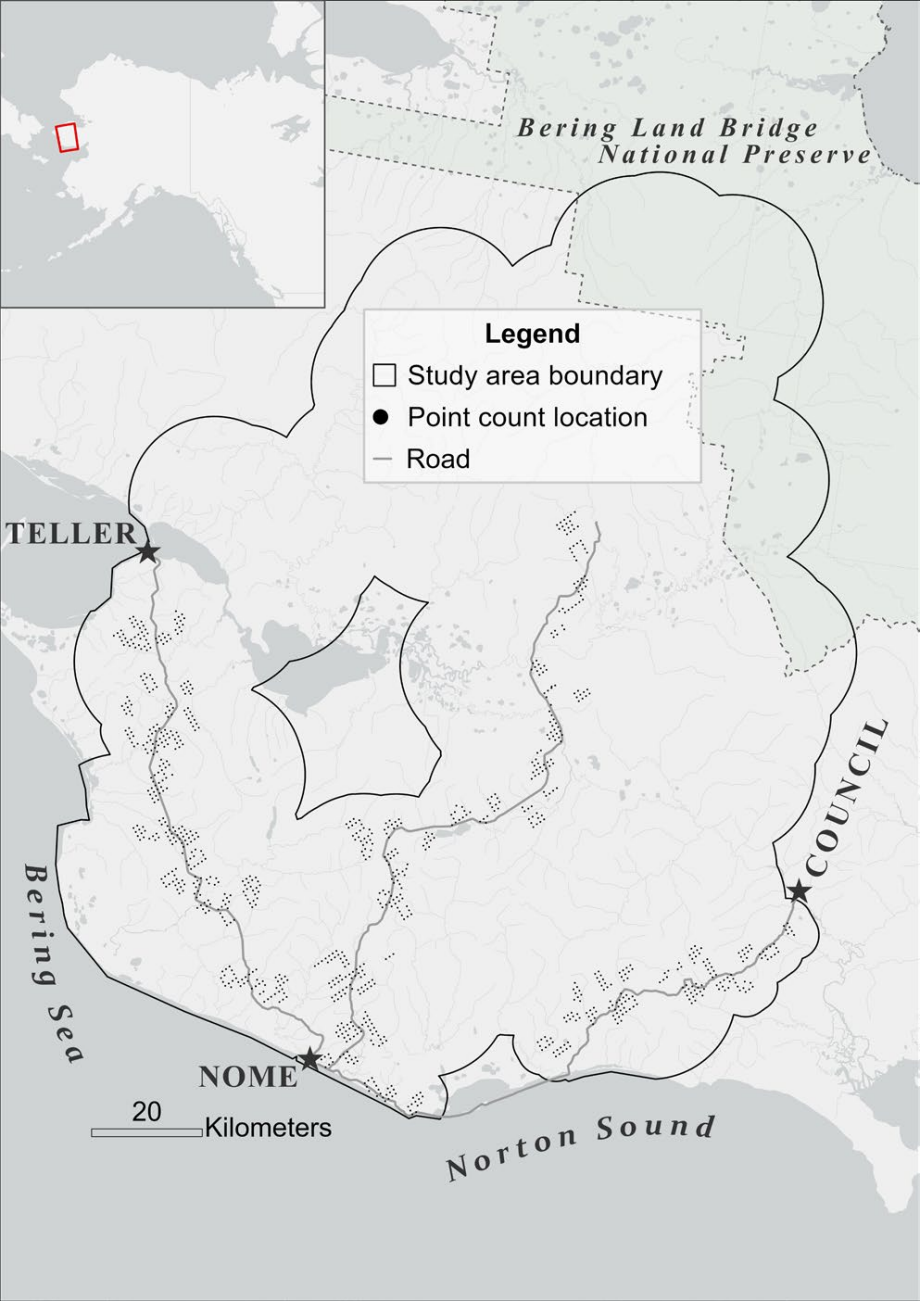
Map of the study area boundary on the southern portion of the Seward Peninsula, Alaska, USA. The inset map shows the location of the Seward Peninsula relative to Alaska (rectangle).

We mapped predictions from our models to an expanded study area based on methods outlined by Anderson et al. (2019). This approach involved merging 15‐km radius buffers around all historical Gyrfalcon territories occupied at least once between 1998 and 2016 with additional 4.5‐km radius buffers around all point count locations surveyed in 2019, 2021, and 2022. This merging ensured that point count survey locations were included in the expanded study area (see Figure 1).

### 2.2 Point Count Surveys

We conducted multi‐species point count surveys (Bibby, Burgess, and Hill 2000; Ralph, Sauer, and Droege 1995) to detect all bird species and Arctic ground squirrels. We commenced surveys 30 min before astronomical sunrise, which is approximately the beginning of civil twilight, or dawn, and ended when at least 10 points were surveyed or ca. 12:00 each day (latest survey at 13:43). Each point was surveyed one time over the duration of 3 years. Surveys used a time‐removal approach where selected time intervals during the survey period act as replicate survey periods (Farnsworth et al. 2002). We conducted surveys for 10 min, preceded by a 2‐min quiet period, and recorded the first detection of an individual (auditory or visual) less than 400 m away (Savard and Hooper 1995; Farnsworth et al. 2002; Matsuoka et al. 2014). Subsequent detections of the same individual (e.g., if a bird continued calling) were not included (Farnsworth et al. 2002). Information collected for each detection included species, time, distance and bearing to individual(s), group size if multiple individuals (≥ 3) were within close proximity (i.e., flocks, squirrel colonies), and sex. Individuals that did not appear to be actively using the area or habitat (e.g., flyovers) were excluded from analysis (Bibby, Marsden, and Jones 1998; Bibby, Burgess, and Hill 2000). A primary observer dictated observations to a recorder.

We conducted point counts from May 10, 2019 to July 23, 2019 (467 points visited), May 5, 2021 to July 16, 2021 (449 points visited), and May 30, 2022 to June 29, 2022 (72 points visited). At each point (site), observers also measured temperature (°C) and wind speed (km/h) using a Kestrel 3000 Wind and Weather Meter and wind direction using a compass. We did not conduct surveys in sustained winds over 24 km/h, or in heavy fog or rain.

### 2.3 Prey Species

Willow Ptarmigan, Rock Ptarmigan, and Arctic ground squirrels are yearlong residents on the Seward Peninsula (Quay 1951; Kessel 1989) although Arctic ground squirrels undergo an extended hibernation period from August to May, depending on sex, age, and other physiological and environmental factors (Sheriff et al. 2012). Willow Ptarmigan are a medium‐sized ground‐dwelling bird in the family Tetraonidae occurring in Arctic, subarctic, and subalpine habitats (Hannon, Eason, and Martin 2020). During the breeding season (April—July), Willow Ptarmigan are typically found in low, moist habitats with dense vegetation, especially willow (*Salix* spp.) or birch (*Betula* spp.) shrub thickets of medium height (0.3–2.0 m) that provide food and shelter (Wilson and Martin 2008; Henden et al. 2011; Ehrich et al. 2012; Kvasnes, Pedersen, and Nilsen 2018; Hannon, Eason, and Martin 2020). They also occur in open tundra, hosting grasses, sedges, tussocks, and low‐growing shrubs (Schieck and Hannon 1993; Kastdalen et al. 2003; Hannon, Eason, and Martin 2020). Rock Ptarmigan share a portion of its range with Willow Ptarmigan but occur farther north than Willow Ptarmigan (Nielsen and Cade 2017; Montgomerie and Holder 2020). Rock Ptarmigan are typically found at higher elevations in dry and rocky habitats with sparse vegetation and dwarf shrubs, and well‐drained grassy tundra (Favaron et al. 2006; Wilson and Martin 2008; Revermann et al. 2012; Pedersen et al. 2014; Hotta et al. 2019; Montgomerie and Holder 2020). The Arctic ground squirrel is a rodent occurring in Arctic and subarctic regions of North America and northeast Russia that exhibit non‐cyclical temporal trends in abundance (ADFG n.d.‐a; Eddingsaas et al. 2004; Faerman et al. 2017). The Arctic ground squirrel inhabits boreal forest, and low‐ and high‐alpine tundra, as well as riverbanks and lakesides, but prefers open alpine meadows with sparse growing low shrubs (e.g., *Dryas* spp.), tall shrubs (e.g., *Salix* spp.), and rocky areas interspersed with forbs and lichens (Batzli and Sobaski 1980; Donker and Krebs 2011).

### 2.4 Habitat Covariates

We obtained land cover data describing vegetation on the Seward Peninsula from the Arctic Boreal Vulnerability Experiment project (hereafter ABoVE; Wang et al. 2019). The ABoVE project classified vegetation groups into 15 types based on the dominant vegetation derived from satellite imagery using a 30‐m grid cell resolution (described in Appendix, Table S1). We used the vegetation dataset from 2014, the most recent year available from the data published by the ABoVE project. Wang et al. (2019) provides further information on data processing, training, and assessments through the Oak Ridge National Laboratory (ORNL) Distributed Active Archive Center (DAAC).

**FIGURE 2:**
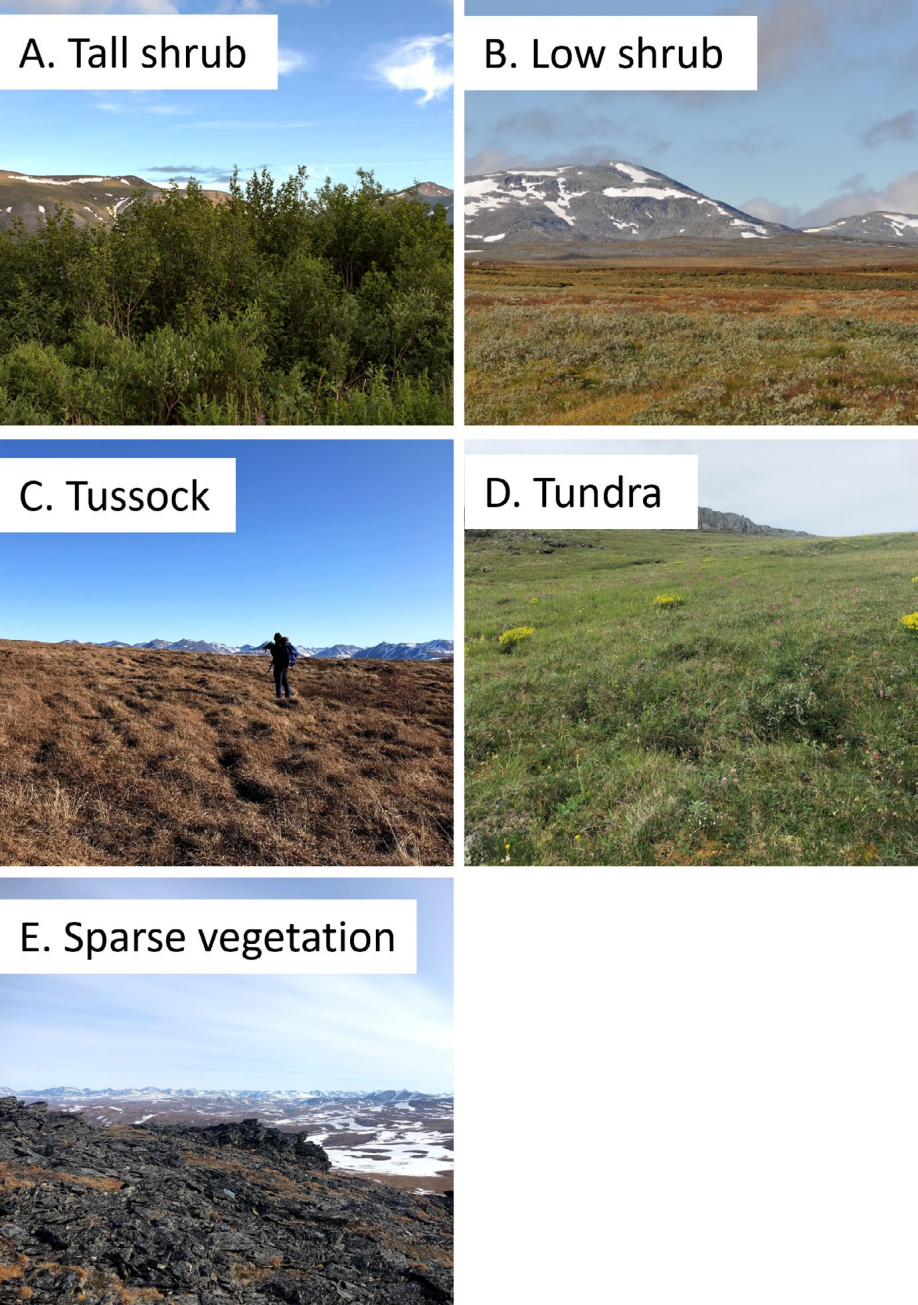
Example photos of vegetation types used in species abundance models. Photo credits: (A) Bill Saltzstein, (B) Mirja Lindberget, (C) Kari Williamson, (D and E) Michaela Gustafson.

For each species, we selected relevant vegetation types and habitat characteristics based on published descriptions of their preferred habitat described above under “Prey Species.” We selected habitats that would be used in each model to avoid correlated predictors and overparametization due to our low sample size for Rock Ptarmigan and Arctic ground squirrels. For Willow Ptarmigan, we selected herbaceous (hereafter referred to as “tundra”), tussock tundra (hereafter referred to as “tussock”), tall shrub, and elevation. For Rock Ptarmigan and Arctic ground squirrels, we selected low shrub, tundra, sparse vegetation, and elevation (examples in Figure 2). We extracted percent cover for each vegetation type within an 800 m × 800 m or 0.64‐km^2^ area surrounding each survey point. We obtained mean elevation within an 0.64‐km^2^ area surrounding survey point using a 5‐m resolution digital elevation model (DEM; U.S. Geological Survey 2017). We selected an area of 800 m × 800 m to closely replicate the area surveyed by point counts. We removed all cells with a mean elevation greater than 500 m as we did not have survey points above this elevation.

Landscape predictors (tundra, tussock, sparse vegetation, low shrub, and tall shrub vegetation types and elevation) were not strongly correlated (Pearson's correlation coefficient, *r*| < |0.70; Akoglu 2018). We standardized landscape predictors by subtracting mean values and dividing by two standard deviations (Gelman 2008; Schielzeth 2010) before they were used in the species models described below. This allowed for direct comparisons of effect sizes and helped model convergence. All data manipulation and analyses were performed using the tidyverse (v2.0.0; Wickham et al. 2019) and unmarked (v1.3.2; Fiske and Chandler 2011) packages in R Statistical Software (v4.1.3; R Core Team 2023).

### 2.5 Gyrfalcon Territory Surveys

We surveyed nesting cliffs in our study area for occupied Gyrfalcon nests in May and June from 2016 to 2022 (except 2020 because of Covid‐19). We monitored 97 Gyrfalcon territories across two surveys per territory each year ranging from May 1 to July 2 during 2016–2022 (except 2020 because of Covid‐19). We conducted surveys from an R44 helicopter following protocols used in Bente (2011) and Anderson et al. (2019). We considered a territory to be potentially occupied if we observed a Gyrfalcon adult, egg, or nestling at a nest during each survey. A total of 60 surveys were not completed because of weather conditions and were assigned as missing values. Territory surveys also occurred every year from 2011 to 2015 but with different protocols. We used these prior surveys to confirm if a territory belonged to a Gyrfalcon during 2011–2015 since some were either unoccupied or occupied by other raptor species during 2016–2022. Our dataset thus, only included territories that were historical Gyrfalcon territories and were monitored during 2016–2022.

### 2.6 Statistical Analysis

#### 2.6.1 N‐Mixture Model for Prey Abundances

We collated detections of unique individuals of the three prey species as repeated counts using a time‐removal design (Farnsworth et al. 2002; Royle 2004). The method involves partitioning the total survey period into time intervals to mimic a removal design. We partitioned counts of unique detections for each species into five, 2‐min intervals. Counts were modeled separately for each species using an N‐mixture, time‐removal model (Kéry and Royle 2016; Chandler 2019) using the “unmarked” package (v1.3.2; Fiske and Chandler 2011; Kellner et al. 2023) in R (v4.1.3; R Core Team 2023). For both ptarmigan species, we retained counts of individuals identified as males in analyses because < 5% of counts for Willow Ptarmigan and < 7% of counts for Rock Ptarmigan were identified as female or unknown sex. We did not count juvenile ptarmigan. Therefore, results for both ptarmigan species are estimates of adult males only. We included all Arctic ground squirrel counts in analyses because sex and age classes are not readily distinguishable in the field. Each species Poisson N‐mixture model consisted of two submodels: (1) relating abundance to landscape predictors, and (2) relating the probability of detecting an individual of the species to predictors that may influence detection. For Willow Ptarmigan, we tested how abundance was related to tall shrub, tundra, tussock, and elevation. For Rock Ptarmigan and Arctic ground squirrel, we tested the relationship between abundance and tundra, low shrub, sparse vegetation, and elevation. Abundance submodels for the three species also included the area sampled by the point counts (assumed to be 0.5024 km^2^ with a 400‐m radius) as an offset, which converted the relative abundance estimated to density values per m^2^ and allowed density estimates to be made for regions of any area (Sillett et al. 2012; Chandler 2019). We did not include year as a factor in the abundance submodels because of low sample sizes. We assumed spatial differences in abundances to be constant and related to habitat. Detection submodels for the three species included ordinal date (recorded as sequential day of year), the number of minutes after civil twilight when the survey began (hereafter referred to as “time of day”), wind speed (kilometers per hour), and observer ID (included as a factor). These predictors have been shown to affect detectability in similar surveys methods (Best 1981; Ralph 1981; Richards 1981; Robbins 1981; Skirvin 1981; Bart and Herrick 1984; Schieck 1997; Simons et al. 2007; Farmer, Leonard, and Horn 2012). We evaluated the significance of predictors in each species model by examining the overlap of the 95% confidence interval with zero. Those predictors having 95% confidence intervals that did not overlap with zero were deemed significant. We also plotted marginalized effect plots for significant predictors. Marginalized effect plots assess the partial relationship between a predictor and the response, while keeping other predictors in the model at a constant value (i.e., the mean).

Lastly, we evaluated model fit for each species model using parametric bootstrapping and Pearson's Chi‐squared statistic (*χ* ^2^) to compare between abundances estimated from the model versus those observed in the data. *p*‐values greater than 0.05 suggest that the observed and estimated abundances are not significantly different from each other and thus indicate that the models are reliable (Kéry and Royle 2016). We also evaluated the dispersion measure ĉ where values > 1 indicate more variance in the observed data than expected by the model and much higher than one (i.e., > 4) poor model fit. A dispersion measure < 1 suggests there may have been more empty sites than what would be predicted by a Poisson model (MacKenzie and Bailey 2004).

#### 2.6.2 Mapping Prey Population Density

We used our estimated abundance models to make predictions of species density on an 800 m × 800 m grid overlapping our study area using the ‘predict’ function from the “unmarked” package (v1.3.2). We excluded cells with mean elevations > 500 m and with covariate values outside the range considered in our prey models. We aggregated the 5‐m digital elevation model using bilinear interpolation to calculate the mean. We standardized each predictor layer (habitat and elevation) with its corresponding mean and two standard deviations from the predictor values used to fit the models.

To estimate prey density inside each Gyrfalcon territory, we excluded cells within each territory with vegetation percentage values that fell outside the ranges used in our prey abundance models, and with elevations exceeding 500 m. We calculated the area size of each territory correcting for these removed cells. We summed expected density of each prey from the grid cells inside each Gyrfalcon territory and divided by the corrected territory area. For Willow Ptarmigan and Rock Ptarmigan, densities were expressed as males per square kilometer, whereas for Arctic Ground Squirrels, densities were reported as squirrels per square kilometer.

#### 2.6.3 Gyrfalcon Occupancy Model

We employed a multi‐season occupancy model that accounts for imperfect detection (MacKenzie et al. 2002) to assess the relationship between annual Gyrfalcon occupancy (during 2016–2019, and 2021–2022) and densities of Willow Ptarmigan, Rock Ptarmigan and Arctic Ground Squirrels inside each territory. The model featured a hierarchical structure with two main components: an ecological submodel that linked occupancy to prey densities and a random intercept for year to allow for annual variability in occupancy, and an observation submodel that related detection probability to the day of the year the survey occurred as well as territory as a random intercept, to account for variability in detection among territories, as well as the repeated measures of territories over multiple years. The short time series and non‐temporal measures of prey density made this model structure more suitable than a dynamic occupancy approach.

We fitted the model using the “unmarked” package (v1.4.1, Fiske and Chandler 2011; Kellner et al. 2023) in R (v4.4.1, R Core Team 2024). We evaluated the significance of predictors based on non‐overlap of 95% confidence intervals with zero. We plotted marginalized effect plots for significant predictors. We evaluated model fit using parametric bootstrapping of Pearson's chi‐squared statistic to compare the tally of observed capture histories against those predicted in the model following MacKenzie and Bailey (2004). This was done using package AICcmodavg (v2.3.3, Mazerolle 2023).

## 3 Results

### 3.1 Summary Statistics

We retained 983 survey points in the analysis after removing points with a mean elevation greater than 500 m.a.s.l. within the surrounding 0.64 km^2^ area. We counted 485 male Willow Ptarmigan at 250 points, 55 male Rock Ptarmigan at 43 points, and 80 Arctic ground squirrels at 42 points (see Table 1 for yearly distribution of counts and how many points for each species).

**TABLE 1:** Yearly distribution of counts for male Willow Ptarmigan, male Rock Ptarmigan, and Arctic ground squirrels and number of points where each species was detected.

**Willow Ptarmigan**		
**Year**	**Individuals counted**	**Number of points**
2019	239	118
2021	237	127
2022	9	5
	485	250
**Rock Ptarmigan**		
**Year**	**Individuals counted**	**Number of points**
2019	31	24
2021	14	13
2022	10	6
	55	43
**Arctic ground squirrel**		
**Year**	**Individuals counted**	**Number of points**
2019	42	17
2021	35	23
2022	3	2
	80	42

*Note:* Prey surveys took place on the Seward Peninsula, Alaska, USA from May through July of 2019, 2021, and 2022.

### 3.2 Prey Distribution and Abundance

We found weak evidence of overdispersion for all three species as indicated by ĉ values that were not significantly higher than 1 (Willow Ptarmigan = 1.01, Rock Ptarmigan = 0.90, Arctic ground squirrel = 0.74), although the Arctic ground squirrel data were underdispersed.

The Pearson's chi‐squared statistic (*χ* ^2^) suggested reasonable fit for the Willow Ptarmigan (*p* = 0.35), Rock Ptarmigan (*p* = 0.62), and Arctic ground squirrel (*p* = 0.60) models.

**FIGURE 3:**
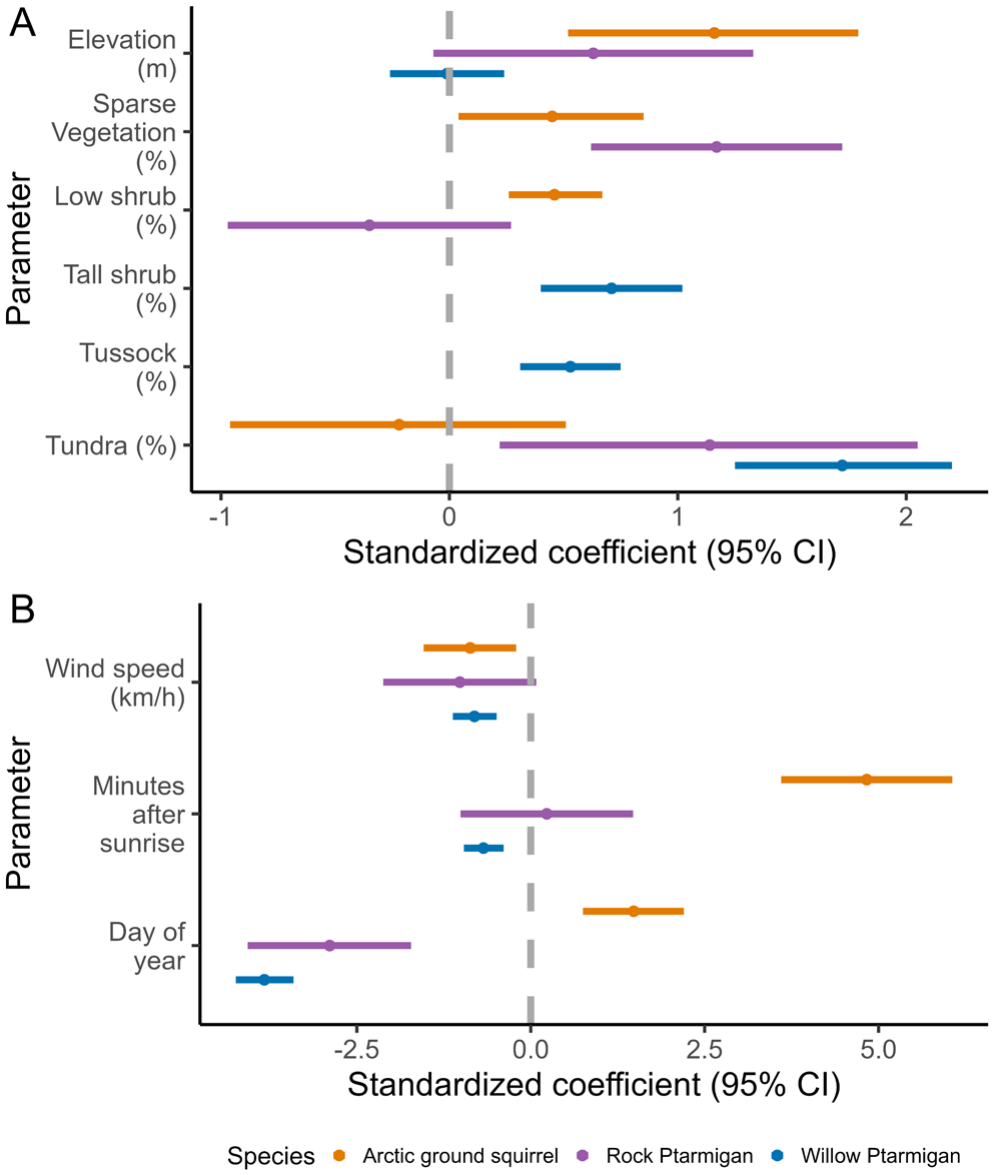
Averaged parameter estimates (standardized regression coefficients) of continuous model predictors are shown as points with lower and upper 95% confidence intervals as horizontal lines for all three species' time‐removal models with abundance (A) and detection (B) submodels. Values that do not overlap with zero (vertical gray dashed line) are considered significant.

**FIGURE 4:**
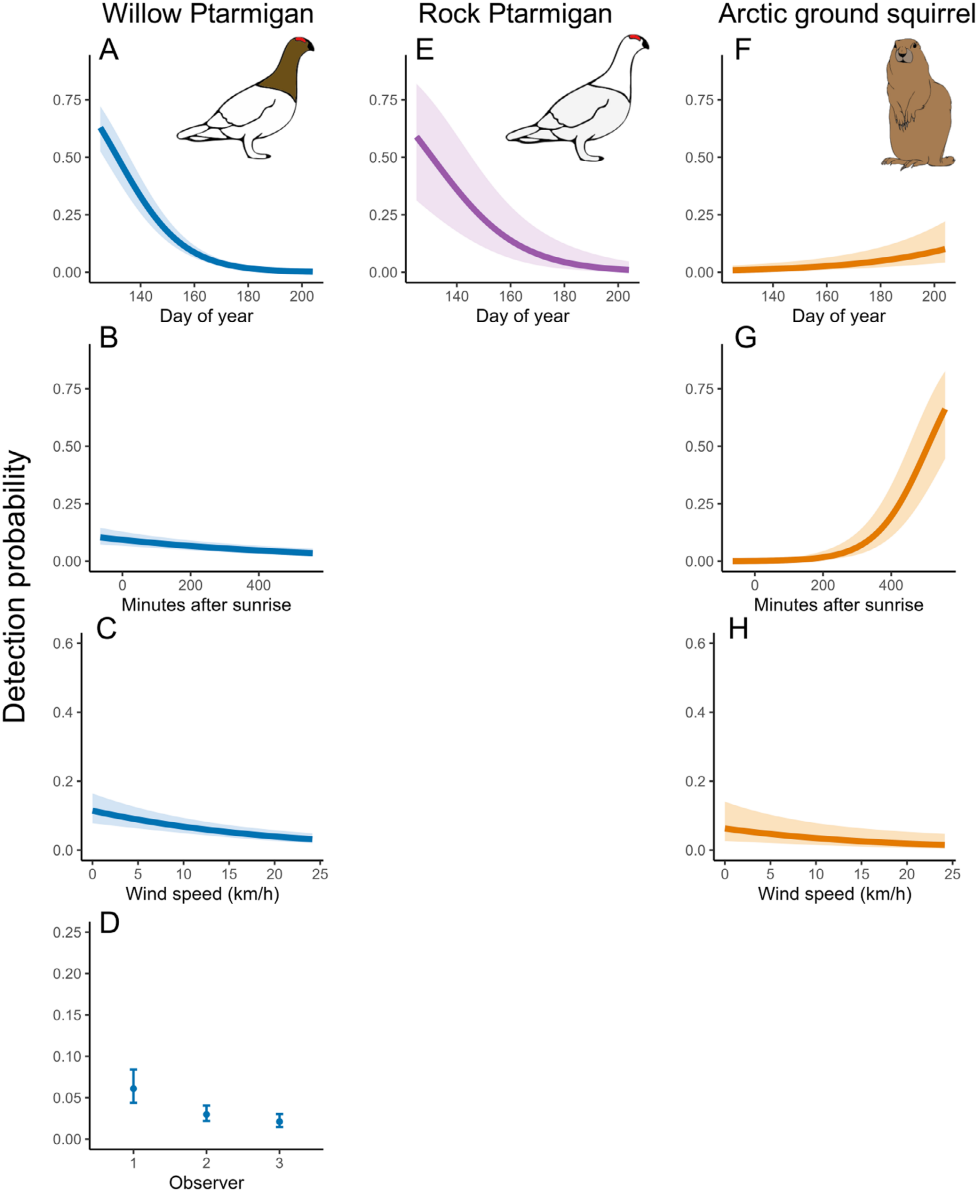
Marginalized effects plot of significant predictors, based on 95% CI, of Willow Ptarmigan (A–D), Rock Ptarmigan (E), and Arctic ground squirrel (F–H) detection on the Seward Peninsula, Alaska, USA from May through July of 2019, 2021, and 2022. Means and 95% confidence intervals are shown by the solid lines and light‐colored bands, respectively. Additional model parameters were held constant.

The probability of detection for Willow Ptarmigan was related primarily to the day of year when surveys were conducted (Figure 3B), with detection declining sharply as the season progressed (Figure 4A). Time of day and wind speed were statistically significant in the detection submodel (Figure 3B) but had weak negative effects on Willow Ptarmigan detection (Figure 4B,C). We found considerable observer variation in detection probability (Figure 3C). Observer detection probabilities are based on a 2‐min temporal replicate within a full 10‐min survey conducted at a given site. Day of year was also important for the probability of detection for Rock Ptarmigan with a negative effect as the season progressed (Figures 3B and 4E). Time of day, wind speed and observer ID had non‐significant effects on the probability of detection for Rock Ptarmigan. Time of day had the strongest relationship with the probability of detection for Arctic ground squirrels (Figure 3B) with detection increasing later in the day (Figure 3G). Day of year and wind speed had significant, but weak, negative effects on detection probability (Figures 3B and 4F,H). Observer ID had non‐significant effects on detection of Arctic ground squirrels.

**FIGURE 5:**
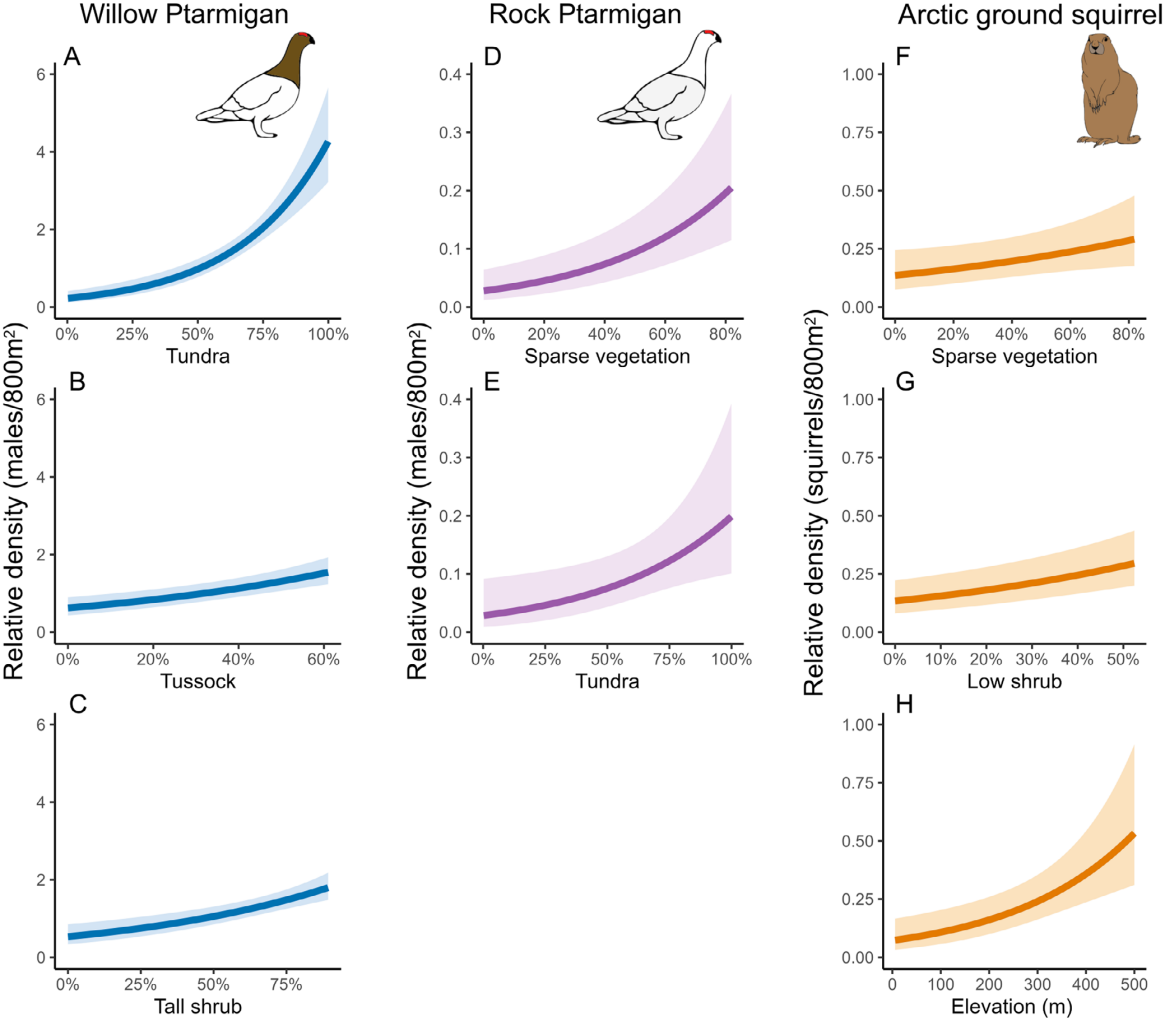
Marginalized effects plots showing the relationship between important (based on 95% CIs non‐overlapping zero) predictors and abundance for (A–C) Willow Ptarmigan, (D–E) Rock Ptarmigan, and (F–H) Arctic ground squirrel. Means and 95% credible intervals are shown by the solid lines and light‐colored shaded area, respectively. Additional model parameters were held at mean values.

Willow Ptarmigan density was significantly and positively correlated to percent cover of tundra (%), followed by the percent cover of tall shrubs and tussock tundra (Figures 3A and 5A–C). Rock Ptarmigan density was significantly and positively correlated with the percent cover of sparse vegetation and tundra (Figures 3A and 5D). Arctic ground squirrel density was significant and positively correlated to cover of sparse vegetation, low shrub, and elevation (Figures 3A and 5F–H).

**FIGURE 6:**
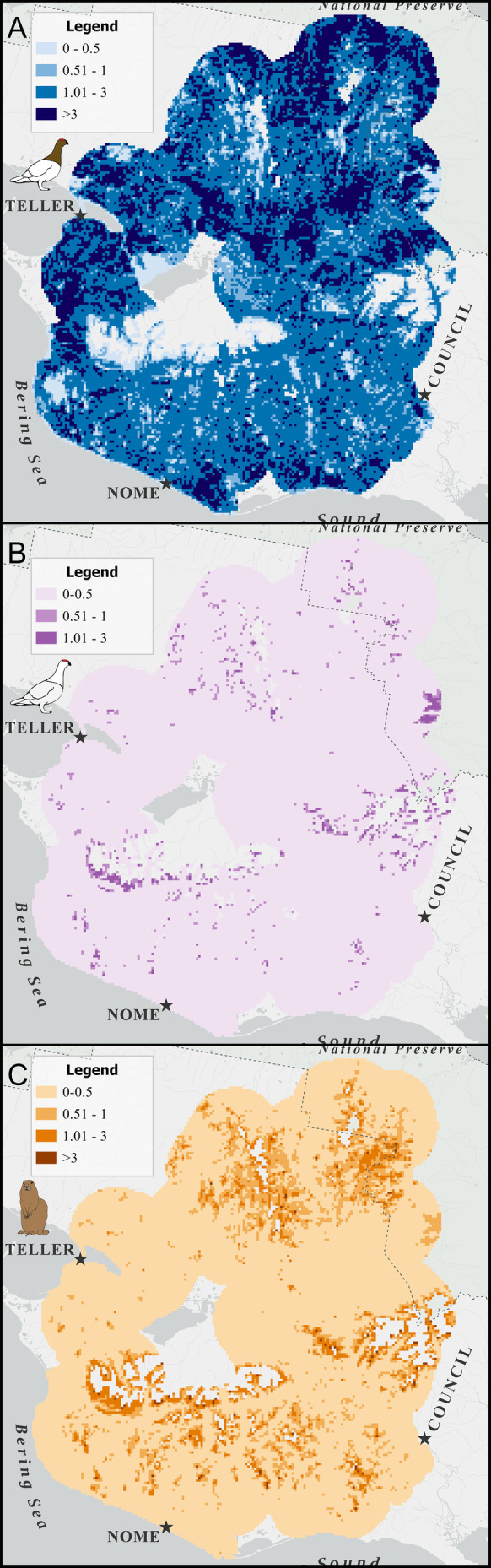
Maps showing the density (males/800 m^2^ for ptarmigan and individuals/800 m^2^ for squirrels) and distribution of three key prey species for Gyrfalcons: (A) Willow Ptarmigan, (B) Rock Ptarmigan, and (C) Arctic ground squirrel, within the study area on the Seward Peninsula, Alaska, USA from May through July of 2019, 2021, and 2022. No densities > 3 were observed for Rock Ptarmigan and that category was not displayed.

Willow Ptarmigan, Rock Ptarmigan, and Arctic ground squirrels differed widely in their spatial abundance and distribution (Figure 6). Willow Ptarmigan were widespread within the study area, with higher densities (> 3 males per 0.64 km^2^) found in the northern portion of the study area, but also more patchily in the west near Teller and in the south near Nome and extending southeast along the Norton Sound (see Figure 6A). The remainder of the study area had densities from 1 to 3 Willow Ptarmigan males per 0.64 km^2^ grid cell making Willow Ptarmigan the most abundant of the three species studied. Willow Ptarmigan had a mean density of 2.13 males per 0.64 km^2^ (95% CI, 1.66–2.76). Minimum and maximum Willow Ptarmigan densities were 0.26 and 5.08 males per 0.64 km^2^ (0.4–7.9 males/km^2^), respectively. Total estimated abundance for the study area was 55,431 males (95% CI, 43,187—71,712).

Rock Ptarmigan were the most sparsely distributed of the three species, with patchy populations scattered across the peninsula, appearing more concentrated in the north and central portions of the study area (see Figure 6B). In areas where Rock Ptarmigan did occur, they were primarily present at densities of < 0.5 males per 0.64 km^2^. Areas with densities > 1 male within 0.64 km^2^ appeared patchily in the north and south, with two areas of higher concentration along the mountains south of Teller and in the northeast within the Bering Land Bridge National Preserve. These high‐density areas were confined to high‐elevation sites within the study area, although elevation was not an important predictor in the model. Rock Ptarmigan had a mean density of 0.15 males per 0.64 km^2^ (95% CI, 0.07–0.33). Minimum and maximum Rock Ptarmigan densities were 0.003 and 2.94 males per 0.64 km^2^ (0.004–4.6 males/km^2^), respectively. Total estimated abundance for the study area was 4041 males (95% CI, 1973—8568).

Arctic ground squirrels were more widespread than Rock Ptarmigan but less abundant than Willow Ptarmigan (see Figure 6C). Areas of high densities occurred in the northern portion of the study area and within the Bering Land Bridge National Preserve and along high elevation areas of the mountains south of Teller extending east to north of Council. Arctic ground squirrels had a mean density of 0.36 squirrels per 0.64 km^2^ (95% CI, 0.21–0.65). Minimum and maximum Arctic ground squirrel densities were 0.07 and 6.65 individuals per 0.64 km^2^ (0.1–10.4 squirrels/km^2^), respectively. Total estimated abundance for the study area was 9512 squirrels (95% CI, 5426—16,965).

### 3.3 Gyrfalcon Occupancy

**FIGURE 7:**
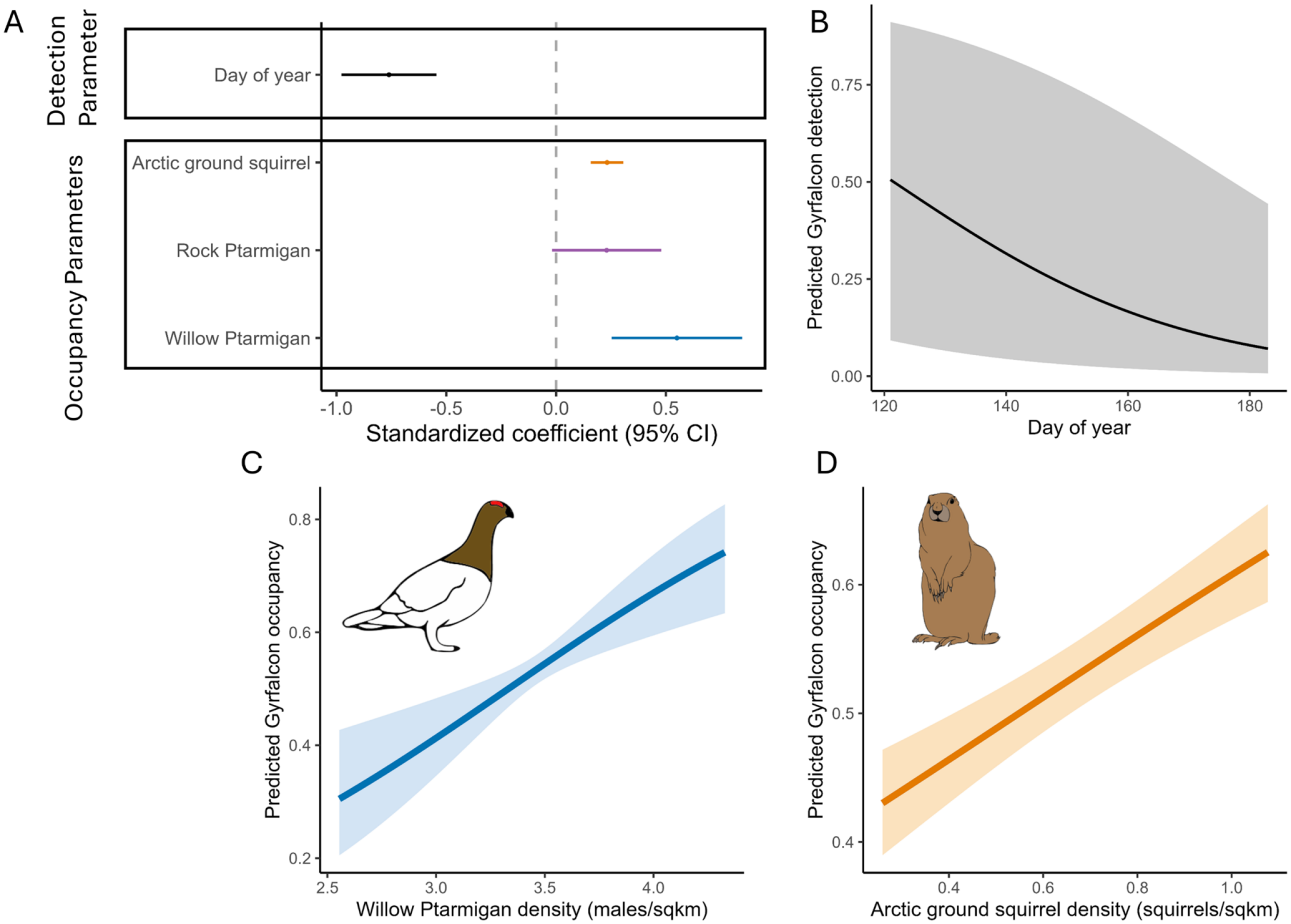
(A, top left) Averaged standardized regression coefficients with 95% confidence intervals for Gyrfalcon occupancy and detection submodels. (B, top right) Marginalized effects plot showing the relationship between day of the year and Gyrfalcon occupancy probability, with mean and 95% credible intervals. (C, bottom left, and D, bottom right) Marginalized effects plots showing the relationship between Willow Ptarmigan and Arctic ground squirrel density and Gyrfalcon occupancy probability, with mean and 95% credible intervals. Other model parameters were held at mean values.

The Gyrfalcon occupancy model was not over‐dispersed (c‐hat = 1.2) and had a reasonable fit based on the *χ* ^2^ statistic (*p* = 0.27). The mean occupancy probability was 0.53 and mean detection probability was 0.53. Gyrfalcon detection probability was higher earlier in the season (Figure 7). Detection also varied largely among territories (variance = 7.46), which reflects the difference in visibility among cliffs. Gyrfalcon occupancy was positively associated with densities of Willow Ptarmigan and Arctic Ground Squirrels (Figure 7A). Rock Ptarmigan density had a marginally significant positive effect on Gyrfalcon occupancy (Figure 7A). Mean Gyrfalcon occupancy probability was ~70% when Willow Ptarmigan density reached 4 males per square kilometer (Figure 7B). Mean Gyrfalcon occupancy probability was ~60% when Arctic ground squirrels reached about 1 individual per square kilometer (Figure 7C). Occupancy did not vary significantly across years (variance = 0.00).

## 4 Discussion

Understanding relationships between Arctic habitats and the abundance and distribution of keystone species or species of conservation concern is a crucial first step in predicting individual responses to habitat shifts resulting from ongoing climate change. We provide the first assessments of habitat‐related abundances of three small herbivore species and one of their top predators on the Seward Peninsula, Alaska, considered fundamental elements of tundra food webs and ecosystem function. We also assessed whether Gyrfalcons were more likely to occupy territories with higher densities these three important prey species. We found Willow Ptarmigan were most abundant in areas with a high proportion of tundra cover, followed by areas with tall shrub and tussock tundra cover. Rock Ptarmigan were most common in areas of tundra and sparse vegetation whereas Arctic ground squirrels were most abundant in higher elevations with sparse vegetation and low shrubs. Rock Ptarmigan and Arctic ground squirrels had patchy distributions, with few pockets of high abundance. Gyrfalcons, in turn, were more likely to occupy territories with higher densities of Willow Ptarmigan and Arctic ground squirrels. Gyrfalcon occupancy was also positively associated with Rock Ptarmigan density, but not significantly.

The habitat relationships for the three species supported previous findings. We found that Willow Ptarmigan were most abundant in areas with a higher percentage cover of open tundra. While tall shrubs and tussock grasses were important, their effect was less pronounced than that of tundra. This supports previous studies suggesting that Willow Ptarmigan rely on a mix of open tundra and shrubs for food, nesting sites, and refugia from predation (Wilson and Martin 2008; Kvasnes, Pedersen, and Nilsen 2018; Hannon, Eason, and Martin 2020). Rock Ptarmigan were most abundant in areas with higher percent cover of open tundra and sparse vegetation, which they likely use for foraging and nesting (Favaron et al. 2006; Wilson and Martin 2008; Sawa, Takeuchi, and Nakamura 2011; Revermann et al. 2012; Hotta et al. 2019; Montgomerie and Holder 2020). Artic ground squirrels were most abundant at higher elevations and with higher percent cover of sparse vegetation and low shrubs, complementing previous studies suggesting that Arctic ground squirrels prefer open habitats for better predator detection (Wheeler and Hik 2014a; Wheeler et al. 2015) and burrowing (Karels and Boonstra 1999).

The three herbivore species are likely to respond differently to shrub encroachment, given their current habitat associations. Increased shrub growth and expansion in Arctic tundra biomes (Tape, Sturm, and Racine 2006; Myers‐Smith et al. 2011; Vuorinen et al. 2017) has largely been attributed to increasing air temperature (Elmendorf et al. 2012; Myers‐Smith and Hik 2018). However, shrub expansion has been heterogeneous and may to be limited by a number of factors such as soil characteristics, topography (Tape, Sturm, and Racine 2006; Myers‐Smith et al. 2011; Tape et al. 2012; Swanson 2015; Ackerman et al. 2017; Liljedahl et al. 2020; Schore et al. 2023), and herbivory (Christie et al. 2015). If shrub expansion remains limited to hillslopes and incised topography such as water tracks, then Willow Ptarmigan will likely benefit. Open tundra and more sparsely vegetated habitats at higher elevations, further from floodplains and riparian zones may be initially resistant to shrub expansion, thereby providing refugia for Rock Ptarmigan and Arctic ground squirrels. However, increased fire frequency and permafrost degradation along with increasing temperatures and summer precipitation may facilitate future shrub growth in areas currently unsuitable for them (Wahren, Walker, and Bret‐Harte 2005; Chen, Hu, and Lara 2021; Liu et al. 2022). Nonetheless, the impacts of Arctic warming and shrub expansion over long time scales remain uncertain and complex, highlighting the need for ongoing monitoring of how animals are responding as these changes occur.

Densities of Willow Ptarmigan (3.32 males/km^2^), Rock Ptarmigan (0.23 males/km^2^), and Arctic ground squirrels (0.56 squirrels/km^2^) on the Seward Peninsula were lower than densities reported elsewhere. Willow Ptarmigan densities ranged between 6 and 229 birds/km^2^ in Norway (Holmstad, Hudson, and Skorping 2005; Kvasnes et al. 2017; Breisjøberget et al. 2018), 9 birds/km^2^ in western Canada, and 7.45–30 birds/km^2^ in Alaska, (Bart et al. 2011). Rock Ptarmigan densities were 2–17 males/km^2^ in Iceland (Nielsen 2011), 8–64 adults/km^2^ in Scotland (Zohmann and Wöss 2008), 0.47–6.4 males/km^2^ in the European Alps (Favaron et al. 2006), and 0.86–5.57 birds/km^2^ in Alaska (Bart et al. 2011). Arctic ground squirrel densities were 50–150 squirrels/km^2^ in northern Alaska (Batzli and Sobaski 1980) 38–610 squirrels/km^2^ in southwest Canada (Donker and Krebs 2011), and 40–270 squirrels/km^2^ in northwestern Canada (Donker and Krebs 2011). Differences in densities across regions may be related to resource availability. Ptarmigan populations in other locations cycle, or go through regular, repeating periods of population increase followed by declines (Fuglei et al. 2020). It is possible our surveys captured populations during a regular period of decline, however, without long‐term population data, we cannot say if the ptarmigan populations on the Seward Peninsula cycle.

Population differences in abundance among regions may also be due to top‐down effects including hunting pressure from humans and predation (Sandercock et al. 2011). In Alaska, Willow and Rock Ptarmigan are managed through hunting regulations, with bag limits of 20 birds per day and seasonal closures (ADFG n.d.‐b). There is no closed season and no bag limits in place for the hunting of Arctic ground squirrels (ADFG n.d.‐a), but Arctic ground squirrels are not a popular or frequently hunted species (Bacon et al. 2009). Rock Ptarmigan populations were highest in Iceland, where major predators include Gyrfalcons, Common Ravens ( *Corvus corax* ), owls ( *Bubo scandiacus* , *Asio otus* , *Asio flammeus* ), Arctic foxes ( *Vulpes lagopus* ), and mink ( *Neovison vison* ). Whereas the Seward Peninsula is host to many avian predators during the breeding season, as well as bears (*Urus arctos*), lynx ( *Lynx canadensis* ), foxes ( *Vulpes vulpes* , *Vulpes lagopus* ), wolverines ( *Gulo gulo* ), ermine or short‐tailed weasel ( *Mustela erminea* ), and least weasels ( *Mustela nivalis* ) that may depredate all three species at various life stages (ADFG n.d.‐c).

Future survey protocols could be modified to maximize detection. Arctic ground squirrels hid in their burrows and Rock Ptarmigan flushed outside the 400‐m boundary as we walked toward the survey points. Aleix‐Mata et al. (2020) show that plot‐sampling methods underestimated Rock Ptarmigan densities by 87%. Thus, alternative survey protocols could include distance sampling or repeated counts from walking transects for Arctic ground squirrels and Rock Ptarmigan (Amundson, Royle, and Handel 2014; Kukka et al. 2021). Further, results from the detection submodels suggest that ptarmigan surveys should continue starting before sunrise and early in the breeding season (April–May), whereas the ground squirrel surveys should shift to starting mid‐morning and surveying throughout the afternoon, later in the season (e.g., July). Due to our low sample sizes for Rock Ptarmigan and Arctic ground squirrels, we caution that our models may not be applicable outside of our study area.

We found evidence that the spatial differences in abundance of the three primary prey species resulted in differences in occupancy among Gyrfalcon territories across the study area. Gyrfalcons were more likely to occupy territories with higher densities of all three species with two being statistically significant. At the population level, Gyrfalcons consume more ptarmigan and squirrels than any other prey item during the breeding season but show shifts in ptarmigan or squirrels being the dominant prey type consumed at different times during the breeding season and from one breeding season to the next (Robinson et al. 2019). However, Gyrfalcons also show individual preferences in their diet with some individuals on the Seward Peninsula having more specialized diets, whereas others ate more diverse diets (Johnson et al. 2022). As shrubs expand throughout the region, the spatial distribution of prey is likely to change. Declines in their primary prey may force predators to shift to alternative species that may not meet their energetic or nutritional requirements (Resano‐Mayor et al. 2016) or may expose them to novel diseases (Radcliffe and Henderson 2023). Overall, changes in habitat are expected to affect prey abundances in different ways. Those changes are expected to scale up the trophic web, impacting the behavior, diet, and demography of the predators that rely on them.

Understanding species abundances and distributions provides a foundation to explore diverse aspects of Arctic ecology, from predator–prey relationships to predicting future spatial changes in habitats and associated species. Recognizing the importance of this insight for conservation and ecosystem functioning, we emphasize the key role of jointly investigating prey abundances, the habitats that they rely on, and the predators that they support. This not only enhances our understanding of Arctic raptor resilience but also contributes to unraveling the complexities of Arctic trophic webs. Our findings extend beyond current knowledge, offering a comprehensive view of habitat dynamics and multi‐trophic web relationships in the Arctic.

## Author Contributions

**Michaela Gustafson:** data curation (equal), formal analysis (lead), investigation (lead), methodology (supporting), supervision (equal), validation (equal), visualization (lead), writing – original draft (lead), writing – review and editing (equal). **Jennifer D. McCabe:** conceptualization (equal), formal analysis (supporting), methodology (equal), writing – review and editing (equal). **Brian W. Rolek:** conceptualization (equal), formal analysis (supporting), methodology (equal), writing – review and editing (equal). **Travis L. Booms:** conceptualization (equal), data curation (equal), funding acquisition (equal), investigation (equal), methodology (equal), project administration (equal), writing – review and editing (equal). **Michael T. Henderson:** data curation (equal), funding acquisition (equal), investigation (equal), methodology (supporting), project administration (equal), supervision (equal), writing – review and editing (equal). **Leah Dunn:** conceptualization (equal), methodology (equal), writing – review and editing (equal). **David L. Anderson:** conceptualization (equal), funding acquisition (equal), methodology (equal), project administration (equal), writing – review and editing (equal). **Jennyffer Cruz:** data curation (equal), formal analysis (supporting), methodology (supporting), supervision (equal), validation (equal), visualization (supporting), writing – original draft (supporting), writing – review and editing (equal).

## Conflicts of Interest

The authors declare no conflicts of interest.

## Declaration of Generative AI and AI‐Assisted Technologies in the Writing Process

During the preparation of this work, the authors used GPT 4.0 (OpenAI) to improve the clarity and readability of this work. After using this tool, the authors reviewed and edited the content as needed and take full responsibility for the content of the publication.

## Supporting information


Appendix S1.

## Data Availability

The data that support the findings of this study are openly available on Dryad. https://doi.org/10.5061/dryad.djh9w0w94.
