# Supplementary material for: Gyrfalcon Prey Abundance and Their Habitat Associations in a Changing Arctic
Source: Ecol Evol. 2025 Jan 8;15(1):e70763. doi: 10.1002/ece3.70763 (PMC11710894; doi:10.1002/ece3.70763)
Supplement: Supplementary file 1 — Appendix S1. [file ECE3-15-e70763-s001.docx]

Appendix S1

Tables

Table S.1 Available land cover types in the Seward Peninsula, Alaska, USA, collected at 30 m resolution from satellite data. Land cover types were derived by the Arctic-Boreal Vulnerability Experiment (ABoVE; Wang et al., 2019). Habitat types included in the abundance submodel of time-removal N-mixture models for each of the three prey species are highlighted by adding the species that was linked to each land cover type in the species model column. We refer to herbaceous as 'tundra', tussock tundra as ‘tussock’, and sparsely vegetated as ‘sparse vegetation’ in the text.

| Land Cover Type | Description | Species model |
| --- | --- | --- |
| Evergreen Forest | Area dominated by tall woody vegetation (> 3m tall) and over 60% canopy coverage with primarily (>75%) evergreen phenological habit (canopy maintains green foliage year-round). |  |
| Deciduous Forest | Area dominated by tall woody vegetation (> 3m tall) and over 60% canopy coverage with primarily (>75%) deciduous phenological habit (annual cycle of leaf-on and leaf-off periods). |  |
| Mixed Forest | Area dominated by tall woody vegetation (> 3m tall) and over 60% canopy coverage with neither forest type (deciduous or evergreen) exceeding over 60% of the area. |  |
| Woodland | Area dominated by tall woody vegetation (> 3m tall) with between 30-60% canopy coverage. Frequently co-exists with peatlands and typically, but not always, evergreen in phenological habit. |  |
| Low Shrub | Area dominated by dense hemi-prostrate to low-erect shrubs (5-30cm in height) with >60% area coverage. Analogous to "prostrate dwarf-shrub" and primarily occurring in tundra areas. | Willow Ptarmigan  Rock Ptarmigan  Arctic ground squirrel |
| Tall Shrub | Area dominated by woody vegetation between 50cm and 3m tall and shrub canopy coverage >60% coverage. Typically, but not always, deciduous phenological habit. | Willow Ptarmigan |
| Open Shrubs | Area with woody vegetation less than 3m tall and between 30-60% canopy coverage. Shrubs typically underlain by herbaceous or barren land cover. | Willow Ptarmigan |
| Herbaceous | Area dominated by herbaceous land cover greater than 60% land cover and tree/shrub cover less than 10%. | Willow Ptarmigan  Rock Ptarmigan  Arctic ground squirrel |
| Tussock Tundra | Tundra-specific herbaceous land dominated by *Eriophorum vaginatum* and other tussock-forming herbaceous species, coverage over 60%. | Willow Ptarmigan |
| Sparsely Vegetated | 10-30% canopy coverage, any vegetation but typically herbaceous/bryophyte, with rock underneath | Rock Ptarmigan  Arctic ground squirrel |
| Fen | Hydrologically connected, sedge/grass dominated wetland |  |
| Bog | Ombrotrophic, peat and shrub dominated wetland |  |
| Shallows/littoral | Lakes <1m deep with some vegetation or shoreline mixed with water/land |  |
| Barren | <10% vegetation, mostly rock |  |
| Water | Oceans, lakes, and rivers, either salt-water or freshwater. |  |

Table S.2 Straight-line distance from point-count survey points to the nearest road and the number of points that fall within that distance category.

| Distance to nearest road | Number of survey points |
| --- | --- |
| 0-200 m | 63 |
| 200m-1 km | 178 |
| 1-2 km | 239 |
| 2-3 km | 193 |
| 3-4 km | 118 |
| 4-5 km | 80 |
| 5-6 km | 64 |
| 6-7 km | 33 |
| 7-8 km | 20 |

Table S.3 Points sampled each year on the Seward Peninsula, Alaska.

|  |  | Number of transects | | Number of points | |
| --- | --- | --- | --- | --- | --- |
| 2019 |  |  |  | |  |
|  | May | 16 | 153 | |  |
|  |  |  |  | |  |
|  | June | 18 | 171 | |  |
|  |  |  |  | |  |
|  | July | 15 | 143 | |  |
|  |  | 49 | 467 | |  |
| 2021 |  |  |  | |  |
|  | May | 20 | 224 | |  |
|  |  |  |  | |  |
|  | June | 14 | 142 | |  |
|  |  |  |  | |  |
|  | July | 10 | 83 | |  |
|  |  | 44 | 449 | |  |
| 2022 |  |  |  | |  |
|  | May | 1 | 8 | |  |
|  |  |  |  | |  |
|  | June | 10 | 64 | |  |
|  |  |  |  | |  |
|  | July | 0 | 0 | |  |
|  |  | 11 | 72 | |  |
|  |  | 104 | 988 | |  |

Table S.4 Mean estimates, standard error (SE), p-values, and upper and lower 95% credible intervals for all three species’ Poisson models with both abundance and detection submodels.

| Variable | Estimate | SE | P-value | 95 CI lower | 95 CI upper |
| --- | --- | --- | --- | --- | --- |
| Willow Ptarmigan | | | | | |
| Abundance |  |  |  |  |  |
| (Intercept) | -3.94 | 0.12 | 0.00 | -4.17 | -3.7 |
| tundra | 1.72 | 0.24 | 0.00 | 1.25 | 2.2 |
| tussock | 0.53 | 0.11 | 0.00 | 0.31 | 0.75 |
| tallshrub | 0.71 | 0.16 | 0.00 | 0.4 | 1.02 |
| elev | -0.01 | 0.12 | 0.95 | -0.26 | 0.24 |
| Detection |  |  |  |  |  |
| (Intercept) observer 1 | -2.73 | 0.18 | 0.00 | -3.08 | -2.39 |
| day of year | -3.83 | 0.21 | 0.00 | -4.24 | -3.41 |
| min after sunrise | -0.68 | 0.14 | 0.00 | -0.96 | -0.39 |
| wind speed | -0.81 | 0.16 | 0.00 | -1.12 | -0.49 |
| Observer 2 | -0.75 | 0.18 | 0.00 | -1.09 | -0.4 |
| Observer 3 | -1.1 | 0.2 | 0.00 | -1.49 | -0.72 |
| Rock Ptarmigan | | | | | |
| Abundance |  |  |  |  |  |
| (Intercept) | -6.5 | 0.28 | 0.00 | -7.05 | -5.95 |
| tundra | 1.14 | 0.47 | 0.01 | 0.22 | 2.05 |
| lowshrub | -0.35 | 0.32 | 0.27 | -0.97 | 0.27 |
| sparseveg | 1.17 | 0.28 | 0.00 | 0.62 | 1.72 |
| elev | 0.63 | 0.36 | 0.08 | -0.07 | 1.33 |
| Detection |  |  |  |  |  |
| (Intercept) observer 1 | -2.11 | 0.48 | 0.00 | -3.05 | -1.16 |
| day of year | -2.89 | 0.6 | 0.00 | -4.07 | -1.72 |
| min after sunrise | 0.23 | 0.63 | 0.72 | -1.01 | 1.47 |
| wind speed | -1.02 | 0.56 | 0.07 | -2.12 | 0.08 |
| Observer 2 | -0.14 | 0.61 | 0.81 | -1.34 | 1.05 |
| Observer 3 | -0.18 | 0.55 | 0.75 | -1.25 | 0.89 |
| Arctic ground squirrel | | | | | |
| Abundance |  |  |  |  |  |
| (Intercept) | -5.53 | 0.21 | 0.00 | -5.95 | -5.11 |
| tundra | -0.22 | 0.37 | 0.55 | -0.96 | 0.51 |
| lowshrub | 0.46 | 0.1 | 0.00 | 0.26 | 0.67 |
| sparseveg | 0.45 | 0.21 | 0.03 | 0.04 | 0.85 |
| elev | 1.16 | 0.33 | 0.00 | 0.52 | 1.79 |
| Detection |  |  |  |  |  |
| (Intercept) observer 1 | -3.45 | 0.45 | 0.00 | -4.33 | -2.57 |
| day of year | 1.48 | 0.37 | 0.00 | 0.75 | 2.2 |
| min after sunrise | 4.83 | 0.63 | 0.00 | 3.6 | 6.06 |
| wind speed | -0.87 | 0.34 | 0.01 | -1.54 | -0.21 |
| Observer 2 | -0.32 | 0.43 | 0.46 | -1.15 | 0.52 |
| Observer 3 | -1.39 | 0.61 | 0.02 | -2.6 | -0.19 |

Figures


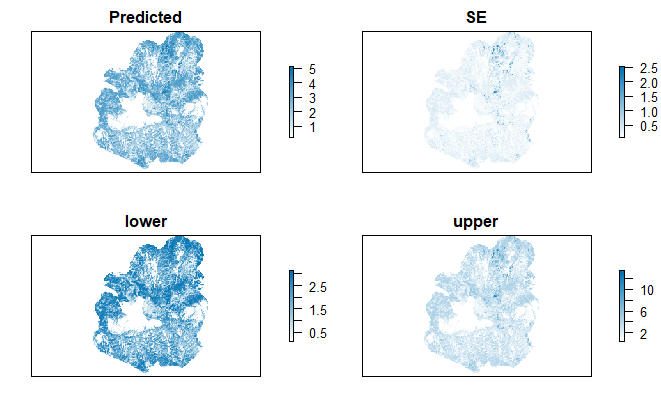


Figure S.1 Estimated mean (panel labeled Predicted), standard error (labeled SE), and 95 % confidence intervals (labeled Lower and Upper panels) densities (males/800m^2^) for Willow Ptarmigan (*Lagopus lagopus*) in the study area located on the Seward Peninsula, Alaska, USA.


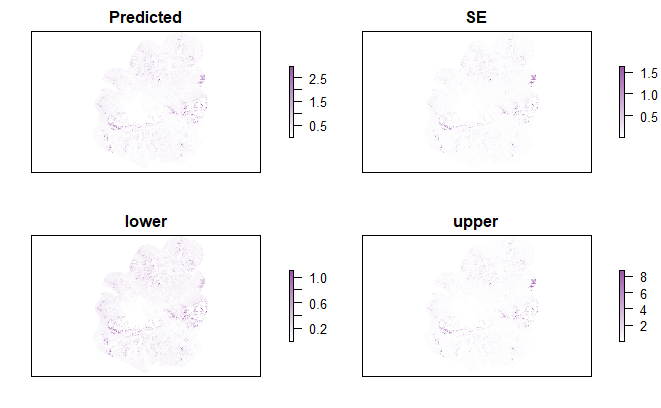


Figure S.2 Estimated mean (panel labeled Predicted), standard error (labeled SE), and 95 % confidence intervals (labeled Lower and Upper panels) densities (males/800m^2^) for Rock Ptarmigan (*Lagopus muta*) in the study area located on the Seward Peninsula, Alaska, USA.


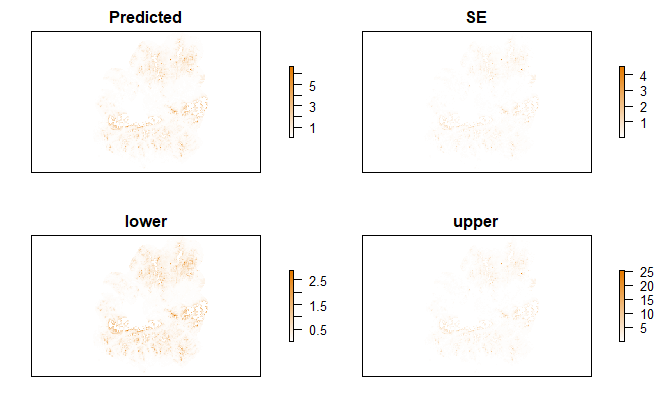


Figure S.3 Estimated mean (panel labeled Predicted), standard error (labeled SE), and 95 % confidence intervals (labeled Lower and Upper panels) densities (squirrels/800m^2^) for Arctic ground squirrel (*Urocitellus parryii*) in the study area located on the Seward Peninsula, Alaska, USA.
